# Supplementary material for: Usefulness of muscle ultrasound in appendicular skeletal muscle mass estimation for sarcopenia assessment
Source: PLoS One. 2023 Jan 17;18(1):e0280202. doi: 10.1371/journal.pone.0280202 (PMC9844922; doi:10.1371/journal.pone.0280202)
Supplement: S1 Table — (DOCX) [file pone.0280202.s001.docx]

**S1 Table. Multiple linear regression analysis (model 4) in men group.**

| Multivariate linear regression, model 4 |  |  |  |
| --- | --- | --- | --- |
| *Variable* | *Β* | *95% CI* | *p-value* |
| Height, cm | 0.228 | 0.152–0.304 | <0.001 |
| Weight, kg | 0.167 | 0.123–0.211 | <0.001 |
| MT of biceps femoris | 0.148 | 0.045–0.252 | 0.004 |
| EI to MT ratio of biceps brachii | -0.822 | -1.649–0.005 | 0.051 |
| Constant | -28.187 | -39.802 to -16.573 | 0.077 |

CI, confidence interval, EI, echo intensity, MT, muscle thickness
