# Supplementary material for: Usefulness of muscle ultrasound in appendicular skeletal muscle mass estimation for sarcopenia assessment
Source: PLoS One. 2023 Jan 17;18(1):e0280202. doi: 10.1371/journal.pone.0280202 (PMC9844922; doi:10.1371/journal.pone.0280202)
Supplement: S2 Table — (DOCX) [file pone.0280202.s002.docx]

**S2 Table. Multiple linear regression analysis (model 4) in women group.**

| Multivariate linear regression, model 4 |  |  |  |
| --- | --- | --- | --- |
| *Variable* | *Β* | *95% CI* | *p-value* |
| Height, cm | 0.215 | 0.183–0.247 | <0.001 |
| Weight, kg | 0.115 | 0.089–0.142 | <0.001 |
| MT of rectus femoris | 0.139 | 0.053–0.226 | 0.002 |
| EI to MT ratio of biceps brachii | -0.638 | -0.904 to -0.372 | <0.001 |
| Constant | -23.502 | -28.185–18.819 | <0.001 |

CI, confidence interval, EI, echo intensity, MT, muscle thickness
