# Supplementary material for: Usefulness of muscle ultrasound in appendicular skeletal muscle mass estimation for sarcopenia assessment
Source: PLoS One. 2023 Jan 17;18(1):e0280202. doi: 10.1371/journal.pone.0280202 (PMC9844922; doi:10.1371/journal.pone.0280202)
Supplement: Attachment — Submitted filename: response to reviewer.docx [file pone.0280202.s003.docx]

**Reviewers' comments**

**Reviewer #1**:

Thanks to the authors for their good job. Sarcopenia is an important issue in aging societies and increasing comorbidities. This article could be a fine guide for the detection and management of sarcopenia. In this respect, I think it will contribute to the literature.

**Response to reviewer #1:**

We appreciate the positive feedback from the reviewer.

**Reviewer #2**

**Comments (1)**

1. Aiming to investigate whether muscle ultrasonography is an effective tool for estimating the appendicular skeletal muscle mass (ASM), the authors combined some parameters, among which some US measurements, into two equations (one for men and one for women). Then, they compared the estimated ASM to the one determined by a BIA analysis. I found this approach questionable as BIA is not the gold standard for ASM assessment. Indeed, ASM is not a BIA direct measure but has to be derived by equation, therefore, its accuracy depends on which equation is adopted by the device. Moreover, other factors influence BIA measurements like hydration status, age, and body mass. About this latter, it has been found that BIA tends to underestimate ASM in overweight people. Even though the equations developed by the authors to estimate ASM took into account age, sex, and body mass, the measure used as reference value cannot be considered equal to the one determined by DXA or CT or MRI. In addition, ASM was estimated by authors feeding the equations with EI values determined during US measurements. Such index, however, has to be considered with cautions as its reliability is age-dependent (Strasser 2013) and it is influenced by several factors among which hydration status, probe position, and probe settings. Even though the reliability of the measure was good, the equations developed by the authors and used to estimate ASM, can be considered valid only in this specific study, with their specific US device, its settings and their operator. These equations on different laboratories may perform differently.

**Response (1)**

We would like to thank the reviewer for their careful reading of this manuscript and valuable comments on this research. We understand your concerns well. As you pointed out, measurement of the exact muscle mass is technically difficult. The most widely used techniques are dual-energy X-ray absorptiometry (DEXA), bioelectric impedance analysis (BIA), computer tomography (CT), and magnetic resonance imaging (MRI). CT and MRI are considered the gold standards for measuring muscle mass measurement because of their high accuracy. However, CT and MRI require expensive equipment for use, which are available only in some research centers or hospitals. In addition, CT carries the risk of exposure to high doses of radiation. In contrast, the DEXA and BIA techniques are relatively inexpensive and easy to use. Recent studies have suggested that BIA can be effectively used to measure muscle mass, including ASM (Kim et al. 2014, Yoshida et al. 2014). BIA has some advantages that can be applied to digital wearable devices, and its validity has been described in previous studies (Ward 2019, Campa et al. 2022). BIA is based on tissue-specific conductivity differences and measures impedance and/or its components: resistance and reactance. However, as you mentioned, BIA methods can be influenced by the individuals' conditions, such as hydration, recent activity, age, sex, and body mass. Recent studies have suggested that multi-frequency BIA methods can reduce the influence of these factors compared to single-frequency (Gába et al. 2015).

In this study, we assessed appendicular skeletal muscle mass (ASM) using multi-frequency BIA methods. All participants underwent multi-frequency 8-electrode BIA methods (InBody 770, Inbody Corp, Seoul, Korea) to assess the ASM. The InBody 770 machine applies a multifrequency alternating current through the body and measures tissue conductivity. Subsequently, body compositions are estimated according to the developed prediction equations using variables of tissue conductivity in combination with other covariates such as sex, weight, and height. The InBody 770 machine had a total of 30 impedance measurements at six different frequencies (1 kHz, 5 kHz, 50 kHz, 250 kHz, 500 kHz, and 1000 kHz) for five body segments (right and left arms, right and left legs, and trunk). In addition, to reduce the influence on their condition, participants were instructed not to eat or exercise for at least three hours before the test and to maintain normal fluid intake the previous day. In the revised manuscript, we have described more detailed methods of muscle mass measurement using BIA in the following sentences:

**(Line 94–98)** *The InBody 770 machine had a total of 30 impedance measurements at six different frequencies (1 kHz, 5 kHz, 50 kHz, 250 kHz, 500 kHz, and 1000 kHz) for five body segments (right and left arms, right and left legs, and trunk). Body composition was estimated according to the developed prediction equations using tissue conductivity variables in combination with other covariates such as sex, weight, and height.*

Another concern of this study is that the developed equation for estimating ASM can be valid only under this specific condition. We agree with your comments. Although ultrasound is a useful tool for evaluating neuromuscular disorders, it is an operator-dependent technique that is affected by different device settings. In addition, muscle echo-intensity values may differ depending on the ultrasound device used. Therefore, the present study was designed to use a single ultrasound device. Accordingly, the developed equation for estimating ASM has some limitations for applications in various ultrasound devices. Nevertheless, the results of our study support the idea that muscle ultrasound could be an effective tool for estimating muscle mass. Further studies using various ultrasound devices are needed to clarify the equation of the estimated ASM according to each ultrasound device.

**References)**

Kim, J. H., S. H. Choi, S. Lim, K. W. Kim, J. Y. Lim, N. H. Cho, K. S. Park and H. C. Jang (2014). "Assessment of appendicular skeletal muscle mass by bioimpedance in older community-dwelling Korean adults." Arch Gerontol Geriatr **58**(3): 303-307.

Yoshida, D., H. Shimada, H. Park, Y. Anan, T. Ito, A. Harada and T. Suzuki (2014). "Development of an equation for estimating appendicular skeletal muscle mass in Japanese older adults using bioelectrical impedance analysis." Geriatr Gerontol Int **14**(4): 851-857.

Ward, L. C. (2019). "Bioelectrical impedance analysis for body composition assessment: reflections on accuracy, clinical utility, and standardisation." Eur J Clin Nutr **73**(2): 194-199.

Campa, F., L. A. Gobbo, S. Stagi, L. T. Cyrino, S. Toselli, E. Marini and G. Coratella (2022). "Bioelectrical impedance analysis versus reference methods in the assessment of body composition in athletes." Eur J Appl Physiol **122**(3): 561-589.

Gába, A., O. Kapuš, R. Cuberek and M. Botek (2015). "Comparison of multi- and single-frequency bioelectrical impedance analysis with dual-energy X-ray absorptiometry for assessment of body composition in post-menopausal women: effects of body mass index and accelerometer-determined physical activity." J Hum Nutr Diet **28**(4): 390-400.

**Comments (2)**

The other limit I found in the study relates to the US ability to discriminate low ASM people. Indeed, after having determined ASM on 212 participants, the authors found that 21 showed low ASM values and, by a ROC analysis, determined a cut-off value on the MT of some muscles. In my opinion the sample size of low ASM group was too poor to return a reliable value.

**Response (2)**

We appreciate your constructive comment. The primary goal of this study was to investigate whether muscle ultrasound could be a tool for estimating muscle mass rather than the ability and cutoff value of US to discriminate low ASM. Healthy participants without weakness or muscle atrophy were recruited for this study. However, some of them had low ASM index (ASM index: <7.0 kg/m^2^ for men and <5.7 kg/m^2^ for women). Therefore, we conducted a subgroup analysis to investigate whether muscle ultrasonography could be used as a screening tool for the low ASM group. We also describe the limitations of our study with a small sample size in the low ASM group. Further studies with a larger sample size for the low ASM group are needed to clarify this issue and the optimal cutoff value for screening sarcopenia.

**MINOR**

**Comments (3)**

In my opinion the introduction is too vague and should be improved, taking care in the terminology. The rationale is not clear and the paragraph dealing with sarcopenia should be delved more deeply. A complete definition of sarcopenia should be given: "Sarcopenia is a progressive, generalized, age-related loss of skeletal muscle mass [1]." is reductive. Notwithstanding the authors continued citing the association to loss of muscle strength and the reduction of physical activity, the official definition of sarcopenia considers sarcopenia as concurrent reduction of skeletal muscle mass, muscle strength and functional physical performance. This latter is different from "physical activity".

**Response (3)**

Thank you for your comments. We have revised the ***Introduction section*** of the manuscript for clarity and conciseness. We also modified "physical activity" to "functional physical performance."

**Comments (4)**

"Historically, muscle mass has been measured using several imaging techniques, including dual energy X-ray absorptiometry, computed tomography, and magnetic resonance imaging." Which of these techniques is considered the gold standard?

**Response (4)**

Several techniques have been used to measure muscle mass. MRI and CT are considered the gold standards for the non-invasive assessment of muscle mass (Mitsiopoulos et al. 1998). However, these techniques are not commonly used in primary clinical practice because they are expensive and require highly trained personnel to use the equipment. In contrast, DEXA and BIA are more widely used for evaluating muscle mass in the clinical field because they are less expensive, easy to assess, and reproducible for estimating ASM.

**References)**

Mitsiopoulos, N., R. N. Baumgartner, S. B. Heymsfield, W. Lyons, D. Gallagher and R. Ross (1998). "Cadaver validation of skeletal muscle measurement by magnetic resonance imaging and computerized tomography." J Appl Physiol (1985) **85**(1): 115-122.

**Comments (5)**

"Muscle ultrasound is a noninvasive, cost-effective, and easily accessible imaging technique for the evaluation of neuromuscular disorders [5]." I find this sentence unnecessary.

**Response (5)**

We deleted this sentence as per your recommendation.

**Comments (6)**

"This study aimed to investigate whether muscle ultrasonography is an effective tool for estimating the appendicular skeletal muscle mass (ASM) in terms of muscle quantity and quality." ASM should be introduced previously, in sarcopenia paragraph otherwise it is not clear why it represents a variable of interest. Moreover, given that the authors previously reported "muscle ultrasonography has been suggested as a valuable tool for assessing sarcopenia because it can assess both muscle quantity and quality" it is not clear which is the novelty of the present study. It seems this study aims to confirm something that is already known.

**Response (6)**

We appreciate your valuable comment. This study was designed to investigate whether ultrasound could be an effective tool for estimating ASM, a representative parameter for muscle mass. Generally, ultrasound is a widely used medical device since it has some merits for being an non-invasive, portable, and less expensive neuroimaging tool. Recently, ultrasound has been extended as a neuroimaging tool for evaluating peripheral nerves and muscles. Muscle ultrasound can be used to assess not only muscle thickness, cross-sectional area, and muscle architecture but also muscle echo intensity. Accordingly, muscle ultrasound could have the potential to evaluate both muscle quantity and quality. Therefore, this study was conducted to investigate whether ultrasound could be applied for estimating muscle mass and screening sarcopenia risk groups in terms of muscle quantity and quality. To clarify the aim of this study, we have revised the paragraph on muscle ultrasound in the ***Introduction*** as follows:

**(Line 61-68)** *Recently, ultrasound has been widely used as an effective neuroimaging tool for assessing the peripheral nerves and muscles. Muscle ultrasound can evaluate muscle thickness, cross-sectional area, muscle architecture, and muscle echo-intensity (EI). Therefore, muscle ultrasound may have the potential to evaluate both muscle quantity and quality. Although several studies have reported the possibility of muscle mass measurement using muscle ultrasound [5-9], it is still not approved as a neuroimaging tool for assessing muscle mass according to the EWGOP and AWGS recommendations [3,4]. In addition, whether muscle EI is valuable for assessing muscle mass and further investigating sarcopenia is rarely reported.*

**Comments (7)**

"In addition, we explored whether muscle ultrasonography can be used as a screening tool for sarcopenia in middle-aged and older individuals" please, explain why you chose such target of population. Is there any reason for involving middle-aged and old individuals?

**Response (7)**

Loss of muscle mass occurs initially in middle-aged individuals. Muscle mass gradually decreases with age (Wilkinson et al. 2018). We believe that it is more meaningful to develop ASM-estimating equations for middle-aged and older individuals. Therefore, we chose this target population (middle-aged and older individuals) for the study.

**References)**

Wilkinson, D. J., M. Piasecki and P. J. Atherton (2018). "The age-related loss of skeletal muscle mass and function: Measurement and physiology of muscle fibre atrophy and muscle fibre loss in humans." Ageing Res Rev **47**: 123-132.

**Comments (8)**

"A hold-out cross-validation method was used to develop and validate the ASM prediction equation." Why did the authors have to validate an ASM prediction equation? The reader has to be previously be informed that US-derived ASM relies on prediction equation. Moreover, the authors should explain the reason why they proposed a different equation.

**Response (8)**

Validation and development are important steps in the development of the prediction model. The hold-out cross-validation method is one of the model evaluation methods, and the dataset is divided into two sets: the training set (development group) and the testing set (validation group). The entire dataset of our study was randomly divided into a model development group (training set) and a validation group (testing set). The ASM prediction equation using ultrasound parameters was then deduced from the model development group, and the accuracy of deducing the ASM equation was verified in the cross-validation group.

**Comments (9)**

Please, do not use WEIGHT but BODY MASS

**Response (9)**

The term "body mass" and "body weight" can be used interchangeably in ordinary conversation. However, these two words do not have the same meaning. "Mass" is the amount of matter in a material, while "weight" is a measure of how the force of gravity acts on that mass. In this study, we measured the "body weight" of each participant. Therefore, we believe that the term "weight" was more appropriate than "mass."

**Comments (10)**

Please, provide an explanation about the test administered in Clinical assessment. Why did they test handgrip and gait speed, and analyzed body composition?

**Response (10)**

We tested hand grip strength to evaluate muscle strength and gait speed to evaluate physical performance. Body composition analysis via BIA methods was performed to measure muscle mass.

**Comments (11)**

Please explain why ASM has been normalized to height.

**Response (11)**

Muscle mass correlates with body size. Therefore, muscle mass-adjusted body size is required to identify the optimal cut-off point for sarcopenia. In previous studies, muscle mass was adjusted for body size in different ways, such as by using height squared, weight, or body mass index. In most cases, the muscle mass adjusted by height squared was used to define the cutoff point. In addition, the EWGSOP and AWGS 2019 consensus have proposed the cutoff point of sarcopenia using ASM normalized with the squared height (Cruz-Jentoft et al. 2019, Chen et al. 2020).

**References)**

Cruz-Jentoft, A. J., G. Bahat, J. Bauer, Y. Boirie, O. Bruyère, T. Cederholm, C. Cooper, F. Landi, Y. Rolland, A. A. Sayer, S. M. Schneider, C. C. Sieber, E. Topinkova, M. Vandewoude, M. Visser and M. Zamboni (2019). "Sarcopenia: revised European consensus on definition and diagnosis." Age Ageing **48**(1): 16-31.

Chen, L. K., J. Woo, P. Assantachai, T. W. Auyeung, M. Y. Chou, K. Iijima, H. C. Jang, L. Kang, M. Kim, S. Kim, T. Kojima, M. Kuzuya, J. S. W. Lee, S. Y. Lee, W. J. Lee, Y. Lee, C. K. Liang, J. Y. Lim, W. S. Lim, L. N. Peng, K. Sugimoto, T. Tanaka, C. W. Won, M. Yamada, T. Zhang, M. Akishita and H. Arai (2020). "Asian Working Group for Sarcopenia: 2019 consensus update on sarcopenia diagnosis and treatment." J Am Med Dir Assoc **21**(3): 300-307.e302.

**Comments (12)**

Please, explain the criteria adopted to choose the muscles that have been measured (why BB, TB, RF ad BF)?

**Response (12)**

In this study, we chose two representative muscles from the upper and lower extremities that are easy to assess using ultrasound. The biceps brachii and triceps brachii are easily accessible muscles in the upper arm. In addition, the rectus femoris (one of the quadriceps femoris muscle groups) and biceps femoris (one of the hamstring groups) muscles are large, thick, and easily accessible in the thigh.

**Comments (13)**

Please, explain why calf muscles have not been considered.

**Response (13)**

This study was designed to evaluate two representative muscles from the upper and lower extremities that are easy to assess using ultrasound and are associated with sarcopenia. Previous studies have reported that sarcopenia was more frequently found in the thigh muscles when compared with calf muscles (Abe et al, 2012, Abe et al, 2014). Thus, we chose thigh muscles for estimating muscle mass and screening sarcopenia. However, regardless of the abovementioned statements, measurement of calf muscle may still be important and a limitation of this study. We described this limitation in the ***Discussion section***.

**(Line 323–327)** *Third, muscle ultrasound data from the distal parts of the arm and leg were not included in this study. Since this study was designed to investigate a simple and quick equation for estimating ASM using muscle ultrasound, only the representative muscles that are easy to access via muscle ultrasound were selected.*

**Comments (14)**

Please, it is recommended to provide all the information about US measurements: gain, frequency of the transducer beam, depth of penetrance, probe length.

**Response (14)**

We added the information in the revised manuscript.

**(Line 105-107)** *Muscle ultrasound was performed using a diagnostic ultrasound device (Aplio i700, Canon Medical Systems, Tochigi, Japan) with an 18-MHz linear probe and B-mode scanning, which was set with Gain 78, a penetrance depth of 3.5 cm, and a probe length of 5.5 cm.*

**Comments (15)**

As ROI appears only two times, I suggest to remove the acronymus.

**Response (15)**

We revised it as per your recommendation.

**Comments (16)**

In the statistical analysis, did the authors check for differences between DEVELOPMENT and cross-validation GROUP?1

**Response (16)**

We did not check for differences between the model development and cross-validation groups. In this study, we used a hold-out cross-validation method. All the data were randomly divided into two groups. The ASM prediction model was then developed using multiple linear regression in the model development group. The developed ASM prediction model was verified by applying it to the cross-validation group. The statistical analysis in this study was performed under the supervision of a biostatistician at our institute.

**Comments (17)**

"Low ASM group was defined as, based on AWGS 2019 consensus, ASM index < 7.0 kg/m2 for men and < 5.7 kg/m2 for women." Please, provide a reference

**Response (17)**

We revised the sentence and added references as follows;

According to the Asian Working Group for Sarcopenia (AWGS) 2019 consensus, the cut-offs of low muscle mass in sarcopenia were defined as <7.0 kg/m^2^ in men and <5.4 kg/m^2^ in women by DEXA and <7.0 kg/m^2^ in men and <5.7 kg/m^2^ in women by BIA [4].

**References)**

4. Chen, L. K., J. Woo, P. Assantachai, T. W. Auyeung, M. Y. Chou, K. Iijima, H. C. Jang, L. Kang, M. Kim, S. Kim, T. Kojima, M. Kuzuya, J. S. W. Lee, S. Y. Lee, W. J. Lee, Y. Lee, C. K. Liang, J. Y. Lim, W. S. Lim, L. N. Peng, K. Sugimoto, T. Tanaka, C. W. Won, M. Yamada, T. Zhang, M. Akishita and H. Arai (2020). "Asian Working Group for Sarcopenia: 2019 consensus update on sarcopenia diagnosis and treatment." J Am Med Dir Assoc **21**(3): 300-307.e302.

**Comments (18)**

Being sarcopenia a combination of muscle mass loss, low muscle strength and reduced functionality, did the authors investigate the correlation between functional parameters and ASM values?

**Response (18)**

We evaluated hand grip strength and gait speed to assess muscle strength and functional physical performance, respectively. Hand grip strength was significantly correlated with ASM and ASM index (ASM/Height^2^). As this study primarily aimed to investigate the utility of muscle ultrasound for estimating muscle mass, we did not include a correlation analysis between functional parameters and muscle mass.

However, we have added a correlation analysis between ASM and clinical parameters as follows **(Line 159–163):** *Pearson’s correlation analysis between the clinical parameters and ASM was performed. ASM was positively correlated with height, weight, BMI, and hand grip strength and negatively correlated with age. In addition, ASM was correlated with muscle thickness but negatively correlated with muscle EI. The results of the correlation analysis are summarized in Table 2*.

**Table 2. Correlation analysis between clinical parameters and appendicular skeletal muscle mass**

|  | Total (n = 212) | | Men (n = 91) | | | Women (n = 121) | | |  |
| --- | --- | --- | --- | --- | --- | --- | --- | --- | --- |
|  | r | p-value | | r | p-value | | r | p-value | |
| Age, years | -0.184 | 0.007 | | -0.527 | <0.001 | | -0.319 | <0.001 | |
| Height, cm | 0.889 | <0.001 | | 0.740 | <0.001 | | 0.751 | <0.001 | |
| Weight, kg | 0.859 | <0.001 | | 0.827 | <0.001 | | 0.741 | <0.001 | |
| BMI, kg/m^2^ | 0.441 | <0.001 | | 0.547 | <0.001 | | 0.374 | <0.001 | |
| Hand grip strength, kg | 0.758 | <0.001 | | 0.470 | <0.001 | | 0.380 | <0.001 | |
| Walking speed, cm/s | -0.012 | 0.860 | | -0.062 | 0.562 | | 0.018 | 0.848 | |
| Muscle thickness |  |  | |  |  | |  |  | |
| Biceps brachii, mm | 0.718 | <0.001 | | 0.327 | 0.002 | | 0.339 | <0.001 | |
| Triceps brachii, mm | 0.525 | <0.001 | | 0.341 | 0.001 | | 0.194 | 0.033 | |
| Rectus femoris, mm | 0.553 | <0.001 | | 0.361 | <0.001 | | 0.234 | 0.010 | |
| Biceps femoris, mm | 0.497 | <0.001 | | 0.393 | <0.001 | | -0.027 | 0.765 | |
| Muscle echo intensity |  |  | |  |  | |  |  | |
| Biceps brachii | -0.299 | <0.001 | | -0.120 | 0.259 | | -0.010 | 0.912 | |
| Triceps brachii | -0.269 | <0.001 | | 0.145 | 0.169 | | 0.057 | 0.535 | |
| Rectus femoris | -0.067 | 0.332 | | -0.075 | 0.478 | | 0.013 | 0.886 | |
| Biceps femoris | -0.235 | 0.001 | | -0.395 | <0.001 | | -0.055 | 0.549 | |

BMI, body mass index

**Comments (19)**

Table 1 To make it more readable, I suggest to use only one column to show mean+/- SD and to thicken the column border of Model development group and cross-validation group

**Response (19)**

We modified Table 1 in the revised manuscript, as you recommended.

**Table 1. Demographic and clinical characteristics of the study participants**

|  | Total (n = 212) | Model development group | | Cross-validation group | |
| --- | --- | --- | --- | --- | --- |
|  |  | **Men (n = 61)** | **Women (n = 90)** | **Men (n = 30)** | **Women (n = 31)** |
|  | Mean ± SD | Mean ± SD | Mean ± SD | Mean ± SD | Mean ± SD |
| **Age, years** | 59.47 ± 10.66 | 61.21 ± 11.40 | 57.88 ± 9.88 | 58.77 ± 12.40 | 61.35 ± 9.13 |
| **Height, cm** | 162.42 ± 8.38 | 169.53 ± 5.66 | 157.27 ± 5.79 | 169.58 ± 6.02 | 156.48 ± 4.98 |
| **Body mass, kg** | 64.64 ± 11.22 | 72.10 ± 10.40 | 57.62 ± 7.19 | 73.63 ± 8.44 | 61.63 ± 9.77 |
| **BMI, kg/m^2^** | 24.39 ± 3.03 | 25.04 ± 3.07 | 23.30 ± 2.63 | 25.56 ± 2.20 | 25.17 ± 3.74 |
| **Hand grip strength, kg** | 26.03 ± 8.68 | 33.36 ± 7.15 | 21.14 ± 4.84 | 32.67 ± 6.46 | 19.35 ± 6.85 |
| **Walking speed, cm/s** | 119.85 ± 18.47 | 119.12 ± 19.59 | 119.38 ± 18.28 | 121.52 ± 19.51 | 121.01 ± 16.30 |
| **ASM, kg** | 18.96 ± 4.55 | 23.10 ± 3.26 | 15.65 ± 2.05 | 23.61 ± 2.88 | 15.91 ± 2.26 |
| **ASM/Height^2^, kg/m^2^** | 7.09 ± 1.09 | 8.01 ± 0.84 | 6.31 ± 0.55 | 8.18 ± 0.63 | 6.48 ± 0.70 |
| **Muscle thickness** |  |  |  |  |  |
| **Biceps brachii, mm** | 13.89 ± 3.04 | 16.36 ± 2.41 | 11.85 ± 1.75 | 16.42 ± 2.96 | 12.53 ± 1.59 |
| **Triceps brachii, mm** | 10.59 ± 3.51 | 12.48 ± 3.96 | 9.12 ± 2.39 | 12.42 ± 3.68 | 9.37 ± 2.58 |
| **Rectus femoris, mm** | 11.26 ± 2.51 | 12.73 ± 2.49 | 10.23 ± 1.95 | 12.53 ± 2.27 | 10.10 ± 2.23 |
| **Biceps femoris, mm** | 19.03 ± 4.53 | 21.34 ± 3.79 | 17.12 ± 3.88 | 22.03 ± 4.51 | 17.10 ± 4.09 |
| **Muscle echo intensity** |  |  |  |  |  |
| **Biceps brachii** | 49.63 ± 2.50 | 48.84 ± 1.88 | 50.11 ± 2.96 | 48.39 ± 1.64 | 51.02 ± 1.62 |
| **Triceps brachii** | 34.99 ± 8.42 | 31.36 ± 8.10 | 37.75 ± 7.50 | 30.81 ± 8.29 | 38.17 ± 7.50 |
| **Rectus femoris** | 45.69 ± 3.96 | 45.37 ± 4.14 | 45.80 ± 3.63 | 45.52 ± 3.16 | 46.19 ± 5.16 |
| **Biceps femoris** | 40.51 ± 5.85 | 41.20 ± 5.49 | 40.81 ± 5.51 | 36.79 ± 7.49 | 41.84 ± 4.39 |

SD, standard deviation; BMI, body mass index; ASM, appendicular skeletal muscle mass.

In addition, we modified Table 5 as presenting with means ± standard deviations

**Comments (20)**

Please, check that measure units are always present.

**Response (20)**

In the revised manuscript, we presented the measure units.

**Comments (21)**

"The estimated ASM did not significantly differ from the measured ASM in either the men or the women groups (p = 0.749 and p = 0.548, respectively; Fig. 2)" Does this sentence relate to MODEL DEVELOPMENT group?

**Response (21)**

Yes, this sentence is related to the model development group. To clarify the meaning, we revised this sentence as follows: **(Line 203–204):** *In the model-development group, the estimated ASM did not significantly differ from the measured ASM in either group: men, p = 0.749; women, p = 0.548 (Fig. 2).*

**Comments (22)**

"without a significant systematic error in the Bland–Altman plot (p = 0.091 and p = 0.056, respectively; Fig. 3)." from which analysis, do these values derive?

**Response (22)**

Thank you for pointing this out to us. To assess the agreement between the measured and estimated ASM, a Bland–Altman plot was drawn. However, we found that these values were derived incorrectly. According to your opinion and our biostatistician’s recommendation, we have deleted these values in the revised manuscript.

**Comments (23)**

Table 4. Differences in the measured and estimated appendicular skeletal muscle mass in the cross-validation group" I would appreciate a Bland_altman plot of these data.

**Response (23)**

We modified and simplified Table 4 as follows:

**Table 4. Intraclass correlation coefficient and standard error of estimated appendicular skeletal muscle mass in the cross-validation group**

|  | ICC (95% CI) | p-value | Standard error (95% CI) |
| --- | --- | --- | --- |
| Men | 0.948 (0.890–0.975) | <0.001 | 0.077 (-0.421–0.574) |
| Women | 0.973 (0.944–0.987) | <0.001 | 0.124 (-0336–0.172) |

ASM, appendicular skeletal muscle mass; CI, confidence interval; ICC, intraclass correlation coefficient.

In addition, we explained the result of paired t-test between the measured and estimated ASM of the cross-validation group in the ***Results section*** as follows: **(Line 207–210)** *The mean value of estimated ASM did not differ significantly from that of measured ASM in either men (23.61 kg vs 23.53 kg, p=0.755; ICC=0.948) or women (15.91 kg vs 15.99 kg, p=0.516; ICC=0.973) in the cross-validation group (Table 4).*

**Comments (24)**

About "Cut-off value in ultrasound-derived parameters for sarcopenia screening" I found questionable diagnosing sarcopenia on ASM alone. Similarly, I found questionable performing statistical analysis on samples with such diversity in size.

**Response (24)**

We do not mean that ASM alone was used to diagnose sarcopenia. As you mentioned, sarcopenia can be diagnosed based on muscle mass, muscle strength, and physical activity. Muscle mass is a key component of the diagnosis of sarcopenia. People with low muscle mass and strength are diagnosed with "sarcopenia." In addition, individuals with low muscle mass, low muscle strength, and low physical performance are diagnosed with "severe sarcopenia". It is presumed that people with low muscle mass are at risk of sarcopenia. To clarify this sentence, we modified it as follows: **(Line 225–226)** *Cutoff value in ultrasound-derived parameters for sarcopenia* ***'risk'*** *screening.* We also considered the small sample size of the low-ASM group, which is a limitation of our study. Therefore, we have mentioned this limitation in the ***Discussion section***.

**Comments (25)**

"Table 5. Comparison between subjects in the normal and low ASM groups." These results come from a statistical analysis that compared groups of different size (within male comparison: 83 vs 8 participants; within female comparison: 108 vs 13 participants). There is no mention in statistical analysis paragraph about this comparison and, in any case, the size is so different that the results lose validity.

**Response (25)**

We have added the following sentence to the statistical analysis paragraph: **(Line 143–147)** *Statistical comparisons between the normal and low ASM groups were performed using independent t-tests for continuous variables.*

In addition, we are also concerned about the small sample size of the low-ASM group, which is a limitation of our study. Therefore, we have mentioned this limitation in the ***Discussion section***. With regard to this limitation, we addressed this issue in our reply to the reviewer's comment #2.

**Comments (26)**

Please add references to: "In addition, muscle ultrasonography has been suggested as a valuable tool for assessing sarcopenia because it can assess both muscle quantity and quality."

"Low ASM group was defined as, based on AWGS 2019 consensus, ASM index < 7.0 kg/m2 for men and < 5.7 kg/m2 for women"(reference is lacking)

**Response (26)**

We added a reference for this sentence.

**(Line 143–145)** *Low ASM group was defined as, based on AWGS 2019 consensus, ASM index of <7.0 kg/m^2^ for men and <5.7 kg/m^2^ for women by employing BIA methods [4].*

**References)**

4. Chen, L. K., J. Woo, P. Assantachai, T. W. Auyeung, M. Y. Chou, K. Iijima, H. C. Jang, L. Kang, M. Kim, S. Kim, T. Kojima, M. Kuzuya, J. S. W. Lee, S. Y. Lee, W. J. Lee, Y. Lee, C. K. Liang, J. Y. Lim, W. S. Lim, L. N. Peng, K. Sugimoto, T. Tanaka, C. W. Won, M. Yamada, T. Zhang, M. Akishita and H. Arai (2020). "Asian Working Group for Sarcopenia: 2019 consensus update on sarcopenia diagnosis and treatment." J Am Med Dir Assoc **21**(3): 300-307.e302.
