# Supplementary material for: Usefulness of muscle ultrasound in appendicular skeletal muscle mass estimation for sarcopenia assessment
Source: PLoS One. 2023 Jan 17;18(1):e0280202. doi: 10.1371/journal.pone.0280202 (PMC9844922; doi:10.1371/journal.pone.0280202)
Supplement: Attachment — Submitted filename: response to reviewers_final.docx [file pone.0280202.s004.docx]

**Reviewers' comments**

**Reviewer #2:**

**Comments (1)**

In some comments I asked you to kindly explain your choices (comment 7, 8, 10, 11, and 12). You carefully addressed the comment in the Response section but, if I've seen correctly, you did not insert the explanation in the manuscript. My recommendations did not aim at receiving an explanation for myself rather providing more details to the readers by adding further explanations in the text. Most of the times I had the answer so I was asking you to improve the manuscript by going deep insight for the readers.

**Response (1)**

We appreciate your careful reading of this manuscript and valuable comments on this research. We revised the manuscript, as you recommended.

1) About your previous comment #7, we modified the following sentence to clarify the purpose of our study and to explain why we included only healthy volunteers aged 41 – 80 years.

**(Line 70-73)** Since muscle mass begins to decline in middle-aged, and decreases gradually with age [10], we explored whether muscle ultrasonography could be used as a screening tool for sarcopenia in middle-aged and older individuals.

2) About comment #8, we added the following sentences to describe a more detailed hold-out cross-validation method in the revised manuscript.

**(Line 82-86)** the entire dataset of our study was randomly divided into a model development group (training set) and a validation group (testing set). The ASM prediction equation using ultrasound parameters was then deduced from the model development group, and the accuracy of deducing the ASM equation was verified in the cross-validation group.

3) About comment #10-12, we modified the manuscript to clarify the methods of this study as follows:

**(Line 95-100)** To evaluate muscle strength, the handgrip strength of the dominant hand was measured using hand-held dynamometry (Jamar hand dynamometry, TEC Inc., Clifton, NJ, USA). To evaluate physical performance, gait speed was measured using gait analysis equipment (GAITRite®, CIR Systems Inc., NJ, USA). To measure muscle mass, body composition analysis was performed via BIA methods using InBody770 (InBody Co. LTD, Seoul, Korea).

**(Line 107-110)** Since muscle mass correlates with body size, muscle mass-adjusted body size is required to identify the optimal cut-off point for sarcopenia. The EWGSOP and AWGS 2019 consensus have proposed the cutoff point of sarcopenia using ASM normalized with the squared height [3, 4].

**(Line 116-123)** In this study, two representative muscles from the upper and lower extremities that are easy to assess using ultrasound were selected. Thus, the biceps brachii (BB) and triceps brachii (TB) in the upper extremity and the rectus femoris (RF) and biceps femoris (BF) in the extremity were chosen for study. MT and EI of these muscles were measured on the dominant hand side.

**Comments (2)**

Table 1 is almost ok. I personally do not appreciate decimals when they lose sense. For example: are two decimals’ essentials when the order of the measure unit is centimeters or millimeters? I understand that the table appears neater when every value is reported with two decimals but I wonder if the second decimals of a millimeter-based value have any sense.

**Response (2)**

In Table 1, all variables were presented as two decimals. Although two decimals may not be essential when the order of the measuring unit is centimeters or millimeters, we presented the two decimals for a unified representation.

**Comments (3)**

In Conclusion I'd suggest a mitigation of the sentence "Muscle ultrasonography can be an effective tool for....." with something like "Muscle ultrasonography seems/appears be an effective tool for..."

**Response (3)**

We modified the sentence in Conclusion, as you recommended,

**(Line 344)** Muscle ultrasonography seems to be an effective tool for estimating muscle mass and screening for sarcopenia.
