# Supplementary material for: Usefulness of muscle ultrasound in appendicular skeletal muscle mass estimation for sarcopenia assessment
Source: PLoS One. 2023 Jan 17;18(1):e0280202. doi: 10.1371/journal.pone.0280202 (PMC9844922; doi:10.1371/journal.pone.0280202)
Supplement: Attachment — Submitted filename: response to reviewers.docx [file pone.0280202.s005.docx]

**Journal Requirements:**

Please review your reference list to ensure that it is complete and correct. If you have cited papers that have been retracted, please include the rationale for doing so in the manuscript text, or remove these references and replace them with relevant current references. Any changes to the reference list should be mentioned in the rebuttal letter that accompanies your revised manuscript. If you need to cite a retracted article, indicate the article’s retracted status in the References list and also include a citation and full reference for the retraction notice.

**Response**

We carefully checked all reference lists and found the #27 article did not correctly include the publication information. So, the information of reference #27 was updated in the revised manuscript as follows:

**(Line 455-458)** 27. Monjo H, Fukumoto Y, Asai T, Ohshima K, Kubo H, Tajitsu H, et al. Changes in Muscle Thickness and Echo Intensity in Chronic Stroke Survivors: A 2-Year Longitudinal Study. J Clin Neurol. 2022;18(3):308-14. <https://doi.org/10.3988/jcn.2022.18.3.308> PMID: 35196746

In addition, this manuscript (PONE-D-22-16915R3) added 1 more reference (Reference #10) to provide further explanations as the reviewer’s recommendation, compared to the previously revised manuscript (PONE-D-22-16915R1). Therefore, the final version of the revised manuscript (PONE-D-22-16915R3) included a total of 27 references. We have reviewed all reference list to ensure that it is complete and correct.
